# Cisplatin based therapy: the role of the mitogen activated protein kinase signaling pathway

**DOI:** 10.1186/s12967-018-1471-1

**Published:** 2018-04-11

**Authors:** Iman W. Achkar, Nabeel Abdulrahman, Hend Al-Sulaiti, Jensa Mariam Joseph, Shahab Uddin, Fatima Mraiche

**Affiliations:** 1 Translational Research Institute, Hamad Medical Corporation, P.O. Box 3050, Doha, Qatar; 2 College of Pharmacy, Qatar University, P.O. Box 2713, Doha, Qatar

**Keywords:** Cisplatin, Mitogen activated protein kinase, p90 ribosomal s6 kinase, Combination therapy, Synergy, Apoptosis

## Abstract

Cisplatin is a widely used chemotherapeutic agent for treatment of various cancers. However, treatment with cisplatin is associated with drug resistance and several adverse side effects such as nephrotoxicity, reduced immunity towards infections and hearing loss. A Combination of cisplatin with other drugs is an approach to overcome drug resistance and reduce toxicity. The combination therapy also results in increased sensitivity of cisplatin towards cancer cells. The mitogen activated protein kinase (MAPK) pathway in the cell, consisting of extracellular signal regulated kinase, c-Jun N-terminal kinase, p38 kinases, and downstream mediator p90 ribosomal s6 kinase (RSK); is responsible for the regulation of various cellular events including cell survival, cell proliferation, cell cycle progression, cell migration and protein translation. This review article demonstrates the role of MAPK pathway in cisplatin based therapy, illustrates different combination therapy involving cisplatin and also shows the importance of targeting MAPK family, particularly RSK, to achieve increased anticancer effect and overcome drug resistance when combined with cisplatin.

## Background

Cisplatin has been widely used since its approval in 1978 against a wide spectrum of tumors including lung, ovarian, testicular, bladder, colorectal and head and neck cancers [1, 2]. Although cisplatin is a widely used drug for cancer chemotherapy, it is also associated with severe dose limiting side effects, and acquired or intrinsic tumor resistance. To circumvent this hindrance, cisplatin has been combined with agents that potentiate the activity of cisplatin towards tumor cells with less toxic side effects [3–6]. The MAPK pathway, including ERK, JNK and p38 kinase, plays a pivotal role in cell survival, proliferation and migration of tumor cells [7–9]. However, targeting these signaling pathways remains controversial as inhibition of this pathway either contributes or prevents cisplatin induced apoptosis. The RSK is a protein which acts as a downstream mediator of MAPK–ERK signaling pathway, and is also associated with cell survival, proliferation, cell cycle progression and migration [10–13]. Although a previous study has shown that RSK inhibition resulted in decreased cell migration and proliferation of cancer cells [14], the effect of combination therapy of cisplatin with RSK inhibition is not clearly understood. Therefore, the aim of this review is to discuss the role of MAPK pathway in cisplatin based chemotherapy and to elucidate the possibilities of combination of RSK inhibition along with cisplatin in order to increase the anticancer effect and reduce resistance and toxic side effects of cisplatin.

## Main text

### Cisplatin

Cisplatin is the first platinum drug approved globally and has been used as a drug for cancer chemotherapy for more than 30 years [1]. Cytostatic property of cisplatin was discovered by Barnett Rosenberg in the late 1960s when he observed anti-proliferative effect of platinum electrodes on bacterial suspension. Cisplatin has gained clinical success and has been proven to target various kinds of cancers, namely, lung, head and neck, bladder, cervical, and ovarian cancers [2].

Upon cisplatin entry to the cell, it becomes activated by hydrolysis. Chloride ions are displaced by water molecules resulting in a strong positive electrophile that covalently binds to any nucleophile such as nitrogen in purine residues. The most reported reaction is the 1,2-intrastrand cross-linkage of the activated cisplatin with the purine residues at nitrogen (N7) which was demonstrated in vitro in DNA of cisplatin-treated salmon sperm cells. These results were further approved in another in vivo experiment that analyzed white blood cells of cancer patients [15, 16]. Cisplatin causes damage in the DNA leading to p53 (tumor suppressor gene) activation. Meanwhile, damaged DNA is subjected to repair via p21 mediated cell cycle arrest. When the damaged DNA is not repairable, p53 induces apoptosis by inhibition of Bcl2 and consequent caspase activation [17]. The main mechanism of cisplatin-induced cancer cell death is via apoptosis. Apoptosis is a procedure of programmed cell death, and is generally manifested by distinct morphological changes to the cell, e.g. cell shrinkage, chromatin condensation, plasma membrane “budding”, exposure of phosphatidyl serine at the cell surface, and caspase activation [18]. The stimulation of a family of cysteines, otherwise known as caspases, is a critical step during the beginning stages of apoptosis. Caspases are characterized as either initiators or executioners of this cell death mechanism. The activation of caspases is dependent upon several stimuli, e.g. caspase-8 (initiator), activated by plasma membrane death receptors (DR), and caspase-9 (initiator), associated with mitochondrial collapse, resulting in caspase-3 and -7 (executioners) activation [19]. Executioner caspases are responsible for many of the biochemical activities contributing towards the induction of apoptosis. This includes poly (ADP-ribose) polymerase (PARP) cleavage and activation, which leads to DNA fragmentation. Apoptosis is carried out by two major pathways known as the extrinsic and intrinsic pathways [20]. The extrinsic pathway begins when ligands bind to tumor necrosis factor-α (TNFα) receptor family leading to the enrollment of procaspase-8 by adaptor molecules, resulting in the development of death-inducing signaling complex (DISC) [21]. However, the intrinsic pathway commences when the cell undergo stress such as DNA damage, which subsequently leads to mitochondrial cytochrome c release and interaction with activating factor-1 (APAF-1), to form an active apoptosome structure, which thereby activates procaspase 9. In response, and in order to regulate DNA damage induced by apoptosis, Bcl-2 family proteins are regulated and undergo several modulations via controlling the release of cytochrome c. Therefore, genotoxic stress induced by cisplatin can result in the activation of several signal transduction pathways, contributing towards the induction of apoptosis.

### Strategies to overcome cisplatin resistance and toxicity

Although cisplatin is widely used anticancer drug, significant challenges still remain in regards to cisplatin resistance and cisplatin induced toxicity. There are different mechanisms attributed towards tumor resistance to cisplatin.

Cisplatin is introduced into cells either through passive diffusion or via transporters (copper transporter, CTR1) [3]. Previous studies have reported that loss of CTR1 resulted in less platinum entering cells and, consequently, drug resistance [22, 23]. Another reason for cisplatin resistance is due to the inactivation of active cisplatin by glutathione or metallothionein present in the cytoplasm of cells. These species are rich in sulphur containing amino acids like cysteine and methionine and leads to inactivation of platinum by binding to sulphur. Active efflux of platinum from the cells through copper exporters (ATP7A and ATP7B) and excretion through glutathione S-conjugate export GS-X pump, also contribute towards cisplatin resistance. Increased DNA repair mechanisms and downregulation of apoptotic pathways are other factors mediating cisplatin resistance [3].

Treatment with cisplatin has been associated with several toxic side effects including nephrotoxicity, oxidative damage to liver, cardiomyopathy, allergic reactions, ototoxicity and gastrotoxicity [15, 24–26].

**Fig. 1:**
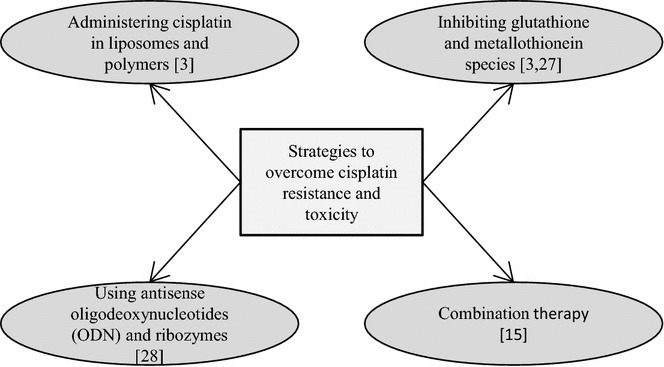
Various strategies to overcome cisplatin resistance. Delivery of cisplatin to target DNA can be improved by administering cisplatin in liposomes or polymers, and by inhibiting cisplatin inactivating species like glutathione and metallothionein. Cisplatin resistance can also be controlled by using antisense oligodeoxynucleotides (ODN) targeting responsible oncogenes for cisplatin resistance or by enhancement of cisplatin activity by combining with other drugs

Cisplatin resistance to tumor cells can be overcome by adopting techniques (Fig. 1) to improve the delivery of cisplatin to the target DNA. This can be achieved by administering cisplatin in polymers or liposomes, and by inhibiting levels of species (glutathione and metallothionein) which inactivate cisplatin and thereby decrease its antitumor efficiency [3]. Recent in vitro and in vivo studies demonstrated that nanoscale micelles encapsulating ethacraplatin, a conjugate of cisplatin, and ethacrynic acid (inhibitor of glutathione S-transferase) resulted in enhanced accumulation of active cisplatin in cancer cells by inhibiting glutathion S transferase and circumventing deactivation of cisplatin [27]. Cisplatin resistance can also be controlled by using antisense oligodeoxynucleotides (ODN) targeting responsible oncogenes for cisplatin resistance, where the ODN hybridize with the target mRNA and prevents its translation; or by inactivation of oncogenes by ribozymes which are RNAs with site specific ligation activity [28].

Owing to treatment resistance and toxic side effects of cisplatin, implementation of combination therapy has gained much importance with regards to increased sensitivity of cisplatin towards cancer cells when combined with other drugs. The combination of other drugs with cisplatin may result in increased anticancer effect, there by result in reducing the dose of cisplatin and ultimately reduce toxic side effects. Hence, combination therapy may be adopted to evade cisplatin toxicity and resistance to cancer cells.

### Combination therapy to overcome resistance and toxicity

#### Cisplatin with chemotherapeutic agents

To overcome the occurrence of resistance observed in patients relapsing from cisplatin treatment, cisplatin is commonly used in combination with various chemotherapeutic drugs and is the basis of many cancer treatments [15]. Cisplatin has been used with a wide range of other cancer drugs for the treatment of ovarian [29], lung [30], breast [31, 32], liver [33], colon [34], prostate [34] and cervical [35] cancers, among many others [15].

Paclitaxel, a pro-apoptotic and mitotic agent, is used to treat numerous cancers including ovarian, lung and breast [15]. Paclitaxel has been shown in combination with 5-fluorouracil and cisplatin for the treatment of advanced gastric carcinoma, and displayed tolerable toxicity in patients [36]. A 2017 phase I trial [37] has also demonstrated that the combination of cisplatin and paclitaxel with triapine, a ribonucleotide reductase inhibitor, can be safely administered together in patients with metastatic or advanced stage solid tumor cancers.

The combination of doxorubicin and cisplatin has been demonstrated to be effective and well tolerated in cancer patients [15]. In vitro anti-tumor studies investigating synergistic cisplatin + doxorubicin combination treatment have been carried out with the aim of overcoming multi-drug resistance [38]. With the use of polymeric nanogels targeting delivery, the combination was effective for the multidrug-resistant MCF-7/ADR tumor and displayed minimal side effects.

In aims to enhance the cytotoxic effects of interferon, a group of natural cytokines displaying antineoplastic effects, specifically in advanced hepatocellular carcinoma [39], interferon was used in combination with other therapeutic agents, including 5-fluorouracil, doxorubicin and cisplatin [40]. The modified combination was associated with an improved response and overall survival in hepatocellular carcinoma patients.

Cisplatin and capecitabine in combination have been demonstrated in gastrointestinal cancers and have shown promising results [41, 42]. However, there remains to be limited data on capecitabine in metastatic breast cancers. A study carried out by Lee et al. [41], demonstrated capecitabine and cisplatin in combination in anthracycline and taxane resistant, heavily pre-treated HER2 negative metastatic breast cancer, displayed clinical benefit with tolerable toxicity in patients. These findings suggest that the proposed combination may offer an option for patients displaying cancer progression after anthracycline and taxane treatment introduction and also showed promise for HR positive metastatic breast cancer patients. Therefore, combination of cisplatin with other chemotherapeutic agents has resulted in increased effectiveness of therapy with reduced side effects.

#### Cisplatin with natural products

Many recent studies reported that combination therapy using natural products, in particular Chinese herbal medicines, is a beneficial therapeutic strategy to overcome the side effects and drug resistance of cisplatin [43–46]. Natural compounds like curcumin improved the efficacy of cisplatin by increasing the sensitivity and inhibition of metastasis by mediating through p21 and cyclin D1 in non-small cell lung cancer cell lines A549 and H2170 [5]. In a study which included an extract of roots of a Chinese herb, Sun Bai Pai, showed apoptosis induction and autophagy by the herbal extract in A549 cells. This effect showed a synergistic action when cisplatin was treated to the autophagic cells indicating that Sun Bai Pai extract improved the cancer cell killing efficiency of chemotherapy drugs even at reduced doses [45]. It suggests that toxic side effects of cisplatin can be reduced in combination therapy. Another example of combination therapy is with melatonin which is an indolamine molecule produced by pineal gland and other organs. Its main function is to control circadian rhythms and to treat insomnia. Melatonin enhanced the cisplatin induced cytotoxicity and apoptosis in SK-LU-1 lung cancer cells as demonstrated by an increase in S-phase arrest [47]. Moreover, combined treatment of melatonin and cisplatin synergistically inhibited the viability of SK-OV-3 ovarian cancer cells. Co-treatment of cisplatin with melatonin increased sub G1 DNA content and TUNEL positive cells compared to cisplatin control, suggesting that melatonin augments the cisplatin induced apoptosis in SK-OV-3 ovarian cancer cells which is consistent to the cleavage of caspase 3 and poly-(ADP-ribose) polymerase (PARP) in the combination treatment. This synergism between cisplatin and melatonin was achieved by inactivation of extracellular signal regulated kinase/p90 ribosomal s6 kinase/heat shock protein 27 cascade in SK-OV-3 cells [44]. Therefore, combination therapy of cisplatin with natural products not only reduced the toxic side effects of cisplatin but also augmented the anticancer activity of cisplatin.

#### Cisplatin with targeted therapeutic agents

Through the advancements made in our knowledge of tumor biology, the vital role of targeted therapies as either first or second line treatment regimens for cancer has been established. The plethora of data accumulated, especially over the last few years, has suggested the promising potential of targeted therapeutic agents use in combination to enhance and support the anti-tumoral effects of conventional chemotherapies [48–50].

Such studies include combination therapy of cisplatin with C-75, which is a selective inhibitor of Fatty Acid Synthase (FASN) [51]. FASN is an enzyme that facilitates de novo synthesis of fatty acids. FASN is overexpressed in various cancers, including epithelial ovarian carcinoma (EOC). This study showed that C-75 in combination with cisplatin caused growth inhibition of EOC tumors in nude mice via FASN down-regulation and caspase activation. Moreover, treatment of EOC cells with C-75 augmented the effect of cisplatin mediated stimulation of apoptosis. Such findings suggest that FASN could potentially be a therapeutic target in this type of cancer, either alone or in combination with chemotherapeutic compounds, including cisplatin to produce synergistic effect [51]. Another example of synergistic effect is demonstrated when cisplatin is combined with PHA665752 [52], a c-Met inhibitor, which resulted in augmented apoptosis of EOC cells. A synergistic inhibition of EOC was also observed in xenograft tumor growth in a nude mouse model, identifying the role of hepatocyte growth factor (HGF)/c-Met pathways in mediating anti-apoptotic signals via AKT in EOC. Furthermore, it has been shown that co-treatment of EOC cells with cisplatin and bortezomib produced more pronounced effect on cell proliferation, apoptosis and S-phase kinase protein 2 (SKP2) down-regulation causing accumulation of p27kip1which is a cyclin-dependent kinase inhibitor [53]. Gleevec (Imatinib) is a FDA approved drug for chronic myelogenous leukemia, where the activity of tyrosine kinase is inhibited by Gleevec by binding to the receptor. Gleevec was also investigated to show its potential activity against cisplatin resistant A549 lung cancer cells. The co-treatment of cisplatin and Gleevec resulted in synergistic cytotoxicity against A549 cells [6] suggesting that combination therapy with Gleevec reduced the resistance of cisplatin against cancer cells. Sorafenib is an approved multikinase inhibitor for the treatment of osteosarcoma. It was found that combination of sorafenib and cisplatin in reduced concentrations considerably reduced the cell proliferation, migration, invasion and colony formation. Furthermore this combination treatment induced cell apoptosis and arrest in the G0/G1 stage of cell cycle in human Saos-2 osteosarcoma cells. The combination also reduced the tumor growth in nude mouse model when compared to individual drug alone suggesting a synergistic growth inhibition [54]. These findings suggest that targeting various cancer promoting pathways along with cisplatin treatment helped to reduce the dose of cisplatin to produce increased anticancer effect. Moreover, reducing the dose of cisplatin diminished the toxic side effects of cisplatin.

#### Cisplatin and inactivation of specific genes

A recent study demonstrated that silencing of YB-1 protein, expressed in various forms of malignant tumors, sensitized SH-SY5Y neuroblastoma cells to cisplatin and stimulated cisplatin induced apoptosis by down regulating multidrug resistance (MDR)1 protein through NFkB signaling pathway [55].

Inactivation of oncogenes responsible for cisplatin resistance is indeed a strategy to improve sensitivity of cisplatin towards tumor cells. In vitro approaches using antisense oligo deoxy nucleotides and by ribozymes (RNAs that have the ability of ligation at particular sites) successfully led to the inactivation of target genes like *c*-*fos, c*-*jun, bcl*-*2, c*-*myc, H*-*ras* etc. resulting in improved activity of cisplatin against cancer cells [28].

An example of combination therapy includes delta-tocotrienol with cisplatin where the combination resulted in downregulation of Notch-1 via inhibition of NFkB signaling pathways resulting in controlling non-small cell lung cancer, while reducing the effective dose of cisplatin [56] suggesting that effective treatment can be achieved with reduced side effects and toxicity of cisplatin.

#### Cisplatin and inhibition of survival pathways

Major survival pathways and their aberrant activation have been reported to frequently occur and contribute towards the development and progression of tumor growth, including the PI3K (phosphatidylinositol 3-kinase)/AKT (protein kinase B)/mTOR (mammalian target of rapamycin) signaling pathway [57–59]. Numerous studies have hence been carried out investigating the interaction between PI3K or mTOR inhibitors and cisplatin, which in fact have displayed a synergistic anti-tumor effect in chemo-naive or resistant cancers, e.g. melanoma, breast, lung and nasopharyngeal cancers [60–63].

Previous studies explored the in vitro effects of mTOR inactivation and evaluated the using of Torin2, an mTOR inhibitor, in EOC cell lines. Sub toxic doses of Torin2 potentiated cisplatin-induced apoptotic activity in EOC. In vivo studies also revealed that in combination of Torin2 and cisplatin, tumor growth in nude mice was synergistically inhibited. Overall, such studies highlight the importance and potential of targeting the mTOR survival pathway, suggesting that co-treatment of cisplatin and mTOR inhibitors, such as Torin2, may be beneficial for the management of EOC and various other resistant cancers [64].

Previous studies have demonstrated that Akt inhibitor, MK-2206, sensitizes cisplatin towards gastric cancer cell line AGS. MTT assay and apoptosis assay demonstrated that cisplatin followed by MK-2206 resulted in synergistic effect of proliferative inhibition and apoptosis respectively for the combination treatment when compared to the monotherapy. The combination treatment also resulted in cleavage of PARP contributing to apoptosis [4]. The Akt inhibitor, MK-2206, also enhanced the cytotoxicity of cisplatin in SK-OV-3 ovarian cancer cells, where consecutive treatment of cisplatin followed by MK-2206 resulted in synergistic inhibition of cell proliferation, enhancement of intracellular reactive oxygen species, and downregulation of pro-survival protein, Bcl-2 [65].

### Cisplatin and mitogen activated protein kinases (MAPKs)

MAPKs are a highly conserved family of structurally related serine/threonine protein kinases responsible for coordinating a variety of extracellular signaling pathways, regulating fundamental cellular processes involved in cell growth and survival [7, 9, 66]. There are three subfamilies in MAPK family, including ERK (member of Ras/MAPK), JNK and p38 kinases [10, 67, 68].

**Fig. 2:**
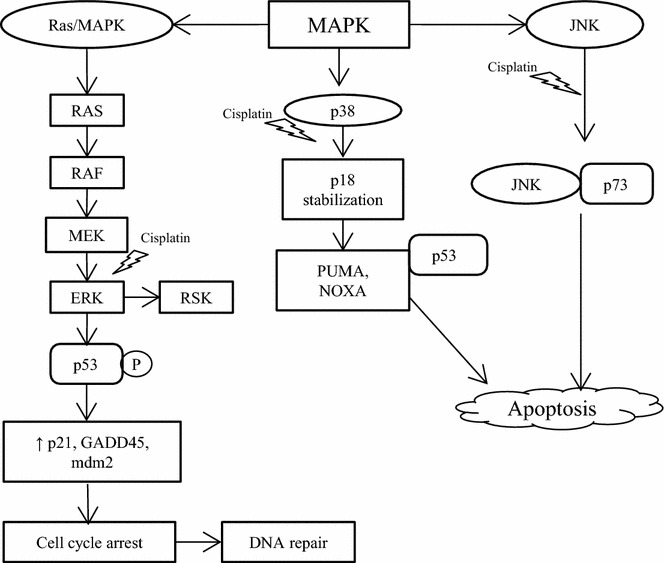
Illustration of MAPK pathway and the role of cisplatin in promoting apoptosis or cell survival. Cisplatin-induced ERK activation phosphorylates p53 which in turn upregulates expression of p21, 45kd-growth arrest and DNA damage (GADD45), and mouse double minute 2 homolog (Mdm2) thereby promoting cell cycle arrest and supporting repair of DNA damage induced by cisplatin. ERK also activates its downstream mediator, RSK which promotes cell survival and metastasis. Cisplatin induced DNA damage activates JNK and p73 and promotes their complex formation and leads to apoptosis. Cisplatin induces stabilization of p18 (Hamlet), a protein regulated by p38 MAPK. This enhances the ability of p53 to interact with and activate pro-apoptotic genes, PUMA and NOXA

Although previous findings have identified cisplatin to result in ERK activation in several forms of cancer, whether this activation prevents or contributes to cisplatin-induced apoptotic effects remains greatly controversial [69–75]. Cisplatin-induced ERK activation precedes p53-mediated DNA damage response as ERK directly phosphorylates p53, resulting in upregulated expression of p21, 45kd-growth arrest and DNA damage (GADD45), and mouse double minute 2 homolog (Mdm2) [76]. As such, ERK activation may result in cell cycle arrest thereby supporting repair of DNA damage induced by cisplatin, via the tumor suppressor p53 (Fig. 2). Furthermore, p53 also directly affects the expression of downstream genes that regulates the sensitivity to apoptosis, activating transcription of *Bax* (promotes apoptosis) and repressing transcription of *Bcl*-*2* (inhibits apoptosis) [28].

Furthermore, DNA damage results in the activation of JNK. The activation of JNK pathway results via both the *cis* and *trans* forms of cisplatin. DNA damage consequently leads to the stabilization and activation of p73, a pro-apoptotic protein related to p53, forming a complex with JNK, mediating apoptosis (Fig. 2) induced by cisplatin [77]. In addition, various other stress stimuli such as environmental stress are important mediators of apoptosis induced via cisplatin. This has been found to occur through the activation of p38 MAPK family. It was reported that cisplatin induces EGFR internalization mediated by p38 MAPK dependent phosphorylation of receptor [78]. Cisplatin induces stabilization of p18 (Hamlet), a protein regulated by p38 MAPK. This enhanced the ability of p53 to interact with and activate PUMA and NOXA, critical pro-apoptotic genes (Fig. 2). Therefore, p38 MAPK pathway plays a crucial part in the regulation of cisplatin ability to induce apoptosis [79]. Moreover, sustained activation of JNK/p38 MAPK pathways by cisplatin has resulted in inducing apoptosis in ovarian carcinoma cells [80]. Previous studies also showed that inhibition of either JNK or p38 kinase attenuated cisplatin induced apoptosis in cervical cancer cells, supporting the role of JNK and p38 in cisplatin response [81]. In contrast, foregoing study has reported that inhibition of p38 MAPK pathway resulted in upregulation of reactive oxygen species, mediating activation of JNK and thereby sensitizing human tumor cells to cisplatin induced apoptosis [82]. In addition, the same study has reported the use of mouse model for breast cancer to confirm that inhibition of p38 MAPK conjoins with cisplatin therapy to decrease tumor size and malignancy [82]. This is also in accordance with another study where p38 MAPK inhibition potentiates cisplatin induced apoptosis via glutathione depletion and drug transport alteration in epithelial renal tubule cell lines [83].

The MAPK cascade also includes upstream signal transducing molecules MEK (MAPK ERK kinase), Raf and Ras. Recent studies have reported the combination of MEK inhibitors along with cisplatin in in vitro and clinical trials [84, 85]. MEK inhibitor along with cisplatin resulted in enhanced apoptotic response in platinum sensitive ovarian carcinoma cells. Phase I/II clinical trials in biliary cancer patients observed significant activity of MEK inhibitor in combination with cisplatin in selected patients with prolonged and complete response to treatment.

### Cisplatin and p90 ribosomal s6 kinase (RSK)

The RSK proteins are the downstream mediators of the Ras–ERK signal transduction pathway. ERK-mediated phosphorylation/activation of RSK plays an important role in regulation of protein translation, cell cycle progression and migration [10, 12, 13]. In addition, RSK proteins have been shown to induce proliferation and survival of cells by enhancing the expression of anti-apoptotic or pro-survival genes, and inhibiting pro-apoptotic genes [11]. A recent research study reported that RSK plays a stimulatory role on ethanol induced hepatocellular carcinoma progression by activating anti-apoptotic factor Bcl-2 and NHE1, known to regulate cell survival [86].

In humans, four RSK isoforms (RSK 1–4) and two structurally related homologs have been identified [87]. Although the RSK isoforms 1–3 are functional, there is a better understanding of RSK1 and RSK2, which display a higher similarity structurally, having a sequence identity of 73%. Furthermore, both isoforms have been associated with tumorigenesis in a wide range of human cancers [88]. More specifically, RSK1, reported to be more frequently activated in melanoma cancer cells, however, were found to be reduced in metastasized lung tissue compared to the primary tumor [8, 89]. Invasiveness and metastasis in head and neck squamous carcinoma cells has been shown to be promoted by RSK2. In addition, RSK2 has reported to contribute towards the survival of multiple myeloma cells [90, 91]. Breast and prostate cancer patients have also displayed higher expression of RSK1 and RSK2 compared to non-cancerous tissue [92, 93]. However, the functions of RSK4 does not resemble to the Ras–ERK pathway. RSK4 has been shown to be involved in p53 mediated cell growth arrest and in oncogene stimulated cellular senescence. The overexpression of RSK4 decreased breast cancer cell proliferation and promoted G0/G1 phase cell cycle arrest. RSK4 has also been reported to be over expressed in greater than 50% of primary malignant lung cancers. RSK3 and RSK4 facilitate resistance to PI3 kinase inhibitors in breast cancer [87]. However, recent research has shown that RSK4 is expressed at low levels in malignant ovarian tumors which correlate with advanced stages of the disease, and cisplatin increases the expression of RSK4 in SKOV3 and TOV112D ovarian cancer cell lines [94]. Therefore, the functions of RSK3 and RSK4 in cancer have not yet been clearly identified.

It is proposed that targeting anti-apoptotic signals associated with the promotion of cell survival may be a promising strategy to optimize the effectiveness of conventional chemotherapeutic agents. RSK mediated signaling involves the phosphorylation and inactivation of pro-apoptotic proteins and the activation of transcription factors and translational machinery that promote cell survival. RSK1 and RSK2 activate pro-survival proteins Bcl-2 and Bcl-xL by post translational phosphorylation and inactivation of the pro-apoptotic protein Bad, enhancing its ability to bind 14-3-3 proteins and preventing its heterodimerization with the pro-survival proteins. RSK1 and RSK2 also phosphorylate CREB leading to increased transcription of Bcl-2 and promote cell survival. Also, RSK1 directly inhibits caspase activity, promoting cell survival. RSK phosphorylates and inhibits GSK3β to promote stabilization of cyclin D1 resulting in cell cycle progression and cell-survival [87]. Previous study has reported that inhibition of RSK with a dihydropteridinone, BI-D1870, which is a potent RSK inhibitor, decreases cell migration and proliferation of A549 human lung adenocarcinoma cells, through phospho GSK 3β [14]. Other inhibitors of RSK include SL0101, LJH685, LJI308 and BIX 02565 which are ATP-competitive inhibitors of the N-terminal kinase domain of RSK, and FMK which is a covalent inhibitor of the C-terminal kinase domain of RSK [10]. Inhibition of RSK2 (ser227) with BI-D1870 induced apoptosis mediated cell death resulting in regressed myeloma cell proliferation [95]. Moreover, combination of BI-D1870 with mTOR inhibitor (everolimus) or histone deacetylase inhibitor (MS-275) or BH3-mimic inhibitor for BCL2/BCLXL (ABT-263), resulted in synergistic or additive anti-proliferative effects in myeloma cells suggesting that RSK2 (ser227) is a potential target in treating patients with multiple myeloma [95]. Furthermore, our own research has demonstrated the effect of combination treatment of BI-D1870 along with cisplatin on migration and apoptosis of A549 lung cancer cells. Our unpublished results indicate that BI-D1870 potentiates cisplatin induced apoptosis and inhibition of metastasis in non-small cell lung cancer. A recent study showed that inhibition of RSK with BI-D1870 overcame the drug resistance to inhibition of Sonic Hedgehog signaling pathway, which has been implicated in the pathogenesis of a variety of human cancers [87]. In a study on ovarian cancer cell line (A2780), treatment with cisplatin downregulated the expression of RSK2. Furthermore, silencing of RSK2 gave rise to improved cisplatin sensitivity [96]. These findings suggest that inhibition of RSK has the potential to sensitize tumors to anticancer agents.

## Conclusion

**Fig. 3:**
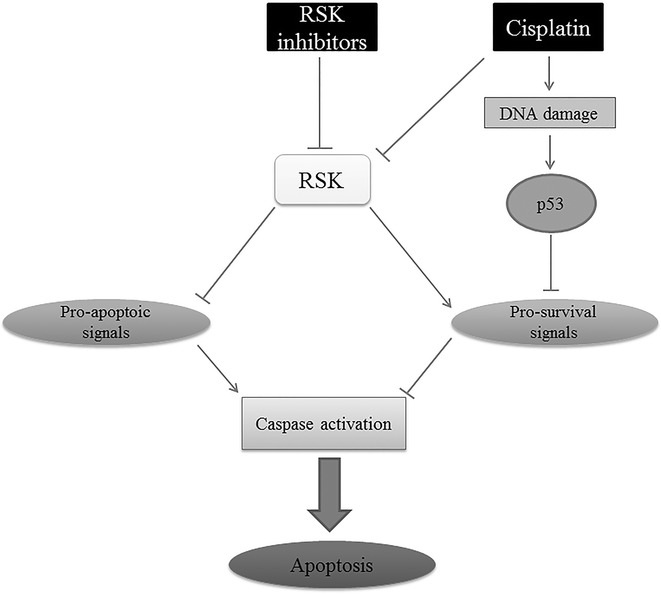
Illustration of combination therapy of cisplatin and RSK inhibitors. Cisplatin causes DNA damage to cell resulting in p53 activation which in turn inhibits pro-survival signals that regress caspase activation. Inhibition of RSK by cisplatin and RSK inhibitors result in inhibition of pro-apoptotic signals there by increasing caspase activation leading to apoptosis. Combination therapy with cisplatin along with RSK inhibitors may result in better inhibition of cancer progression

This review article depicted the importance of combination therapy in treatment of cancer. Standard chemotherapy with cisplatin has limitations, such as drug resistance and its toxic side effects. However, these limitations can be overcome by administering low doses of cisplatin, which can be achieved in combination with such agents which would increase the sensitivity of cisplatin towards cancer cells. Studies conducted in recent years suggested the importance of administering targeted therapeutic agents in combination with cisplatin to enhance the anti-tumoral effects of cisplatin. It would be beneficial to target the major cell survival pathways that are upregulated in various types of cancers. The MAPK family, including its members ERK, p38, JNK and downstream mediator, RSK, executes major cellular functions like proliferation, survival and differentiation. However, the inhibition of ERK, p38 and JNK either prevents or contributes towards cisplatin induced apoptosis remains controversial; which necessitates focusing on inhibition of RSKs which are the downstream effectors of the ERK/MAPK signaling cascade. RSKs are implicated in various cellular processes and modulate cell proliferation, tumorigenesis and metastasis in various cancers. Therefore, as shown in Fig. 3, cisplatin causes DNA damage resulting in p53 activation leading to inhibition of pro-survival proteins and consequent caspase activation and apoptosis. On the other hand, inhibition of pro-apoptotic signals by RSK is controlled and limited by RSK inhibitors, there by promoting apoptosis. It is also worthy to note that RSK isoforms (RSK 1–4) in different tissues varies in their expression levels. RSK1 is expressed predominantly in the lung, kidney and pancreas [97]. RSK2 and RSK3 are highly expressed in heart, skeletal muscle and pancreas [97, 98]. RSK4 expression is much lower when compared with other RSKs. Previous study on mice demonstrated that RSK4 is expressed in brain, heart, kidney and skeletal muscle, whereas in lung, adipose tissue, liver and pancreas, RSK4 expression was not detected [97, 99]. This suggests that the effectiveness of RSK inhibitors used in combination therapy with cisplatin is also determined by the type of cancer based on tissues and organ.

Although this review focused on cisplatin, the findings discussed here are not only specific to cisplatin but also applicable to a class of platinum derived chemotherapeutic drugs. Platinum derived agents like carboplatin and oxaliplatin are other globally approved anticancer drugs and have similar mechanism of action as cisplatin [1, 2]. Carboplatin is a second generation platinum drug which is used in combination with paclitaxel against ovarian cancer [2, 100]. In vitro studies have proved synergistic effect of carboplatin in combination with cytosine arabinoside and mitoxantrone against human leukemia cell lines [101]. Previous studies have also demonstrated synergistic antitumor efficacy of carboplatin and trastuzumab in metastatic breast cancer [102]. A recent study has demonstrated that the HSP90 inhibitor, ganetespib synergizes the antitumor efficacy of carboplatin in ovarian cancer cells in vitro and tumor xenografts in vivo [103]. Moreover, a recent study has demonstrated that combination of carboplatin with either everolimus (mTOR inhibitor) or trametinib (ERK inhibitor) decreased cellular proliferation of astrocytoma cells [104]. Furthermore, everolimus sensitized astrocytoma cells to carboplatin treatment, despite astrocytoma cells being resistant to everolimus treatment alone. These findings together suggested that combination therapy increased the anticancer activity and overcame drug resistance. Other platinum derived drugs such as nedaplatin and lobaplatin have been used in combination therapy in clinical trials of metastatic oesophageal carcinoma [1]. Heptaplatin is another platinum derived drug applied in clinical trials in combination therapy for head and neck cancer [1]. The newly developed platinum based drugs were to reduce the resistance and toxicity caused by cisplatin. Carboplatin was preferred as the platinum agent of choice over cisplatin in advanced ovarian cancer in palliative settings owing to its less nephrotoxicity and neurotoxicity when compared to cisplatin [105]. However combination therapy based on carboplatin was found to be inferior to that based on cisplatin [105]. This suggests that cisplatin is still a drug of choice in combination therapy for cancer treatment.

In conclusion, targeting MAPK pathway predominantly in its downstream signaling cascade would be favorable in cancer treatment. A combination therapy of platinum based drugs with a RSK inhibitor would be a beneficial strategy to overcome the resistance and toxic side effects of platinum drugs and ultimately to combat cancer.

## Abbreviations

CTR1: copper transporter 1
EOC: epithelial ovarian carcinoma
ERK: extracellular signal regulated kinase
FASN: fatty acid synthase
JNK: c-Jun N terminal kinase
MAPK: mitogen activated protein kinase
PARP: poly (ADP) polymerase
RSK: ribosomal s6 kinase
